# Purpose-Dependent Consequences of Temporal Expectations Serving Perception and Action

**DOI:** 10.1523/JNEUROSCI.1134-20.2020

**Published:** 2020-10-07

**Authors:** Freek van Ede, Gustavo Rohenkohl, Ian Gould, Anna C. Nobre

**Affiliations:** ^1^Oxford Centre for Human Brain Activity, Wellcome Centre for Integrative Neuroimaging, Department of Psychiatry, University of Oxford, Oxford OX3 7JX, United Kingdom; ^2^Department of Experimental Psychology, University of Oxford, Oxford OX2 6GG, United Kingdom; ^3^Institute for Brain and Behavior Amsterdam, Department of Experimental and Applied Psychology, Vrije Universiteit Amsterdam, 1081BT, Amsterdam, The Netherlands

**Keywords:** alpha oscillations, attention, behavioral performance, beta oscillations, task-dependent, temporal expectation

## Abstract

Temporal expectations enable anticipatory brain states that prepare us for upcoming perception and action. We investigated the purpose-dependent nature and consequences of cued temporal expectations on brain and behavior in male and female human volunteers, using two matched visual-motor tasks that stressed either response speed or visual accuracy. We show that the consequences of temporal expectations are fundamentally purpose dependent. Temporal expectations predominantly affected response times when visual demands were low and speed was more important, but perceptual accuracy when visual demands were more challenging. Using magnetoencephalography, we further show how temporal expectations latch onto anticipatory neural states associated with concurrent spatial expectations—modulating task-specific anticipatory neural lateralization of oscillatory brain activity in a modality- and frequency-specific manner. By relating these brain states to behavior, we finally reveal how the behavioral relevance of such anticipatory brain states is similarly purpose dependent.

**SIGNIFICANCE STATEMENT** Knowing when events may occur helps to prepare neural activity for upcoming perception and action. It is becoming increasingly clear that distinct sources of temporal expectations may facilitate performance via distinct mechanisms. Another relevant dimension to consider regards the distinct purposes that temporal expectations may serve. Here, we demonstrate that the consequences of temporal expectations on neurophysiological brain activity and behavior are fundamentally purpose dependent, and show how temporal expectations interact with task-relevant neural states in a modality- and frequency-specific manner. This brings the important insight that the ways in which temporal expectations influence brain and behavior, and how brain activity is related to behavior, are not fixed properties but rather depend on the task at hand.

## Introduction

Temporal expectations help us prepare for upcoming perception (Jones and Boltz, 1989; Coull and Nobre, 1998; Ghose and Maunsell, 2002; Lange and Röder, 2006; Lakatos et al., 2008; Jaramillo and Zador, 2011; Lima et al., 2011; Rohenkohl et al., 2012; Vangkilde et al., 2012; Meijer et al., 2016; Auksztulewicz et al., 2018) as well as action (Riehle et al., 1997; Shin and Ivry, 2002; Janssen and Shadlen, 2005; Schoffelen et al., 2005; Praamstra et al., 2006; van Elswijk et al., 2007; Los et al., 2017; Heideman et al., 2018b, 2020; Boettcher et al., 2020). It is increasingly recognized that distinct sources of temporal expectations, such as associations, rhythms, hazard rates, and sequences (Nobre and van Ede, 2018), may facilitate performance via distinct neural mechanisms (Rohenkohl et al., 2011; Triviño et al., 2011; de la Rosa et al., 2012; Breska and Deouell, 2014; Morillon et al., 2016; Breska and Ivry, 2018; Nobre and van Ede, 2018; Bouwer et al., 2020). In addition to distinct sources of temporal expectation, another relevant dimension to consider regards the distinct purposes that temporal expectations may serve (Shalev et al., 2019). Is it possible for the same type of temporal expectation to have distinct consequences on brain activity and on behavioral performance depending on the anticipated task demands?

To address this, we used a classic associative temporal orienting task (Coull and Nobre, 1998; Nobre, 2001), in which cues predict the time at which a target will appear, and measured neural anticipation using magnetoencephalography (MEG). Critically, we varied whether the anticipated task stressed motor demands, by requiring a speeded response to an easy-to-discriminate visual target (“motor task”), or stressed perceptual demands, by requiring a difficult visual discrimination (“visual task”). In both tasks, cues also provided valid spatial foreknowledge about the relevant response hand (motor task) or visual target location (visual task). This allowed us to assess the influence of temporal expectations in the presence of concurrent spatial expectations (Doherty et al., 2005; Rohenkohl et al., 2014) and to ask whether temporal expectations modulate purpose-specific electrophysiological signatures of spatial anticipation in a modality- and frequency-specific manner.

## Materials and Methods

Data from the visual task has previously been published in the context of an aging study where we compared these data to those from a group of older participants doing the same task (Heideman et al., 2018a). The central aspect of the current article involves the comparison between the visual task and the matched motor task in which the same participants took part. Tasks were matched in that we used identical spatial-temporal cues and cue validities in both tasks (while varying task demands between tasks). Neither the data from the current motor task nor their comparison to data from the visual task have been published previously.

### 

#### Participants

Twenty right-handed human volunteers (7 female; age range, 18–33 years; mean age, 24.1 years) participated in both the motor task and the visual task. All participants had normal or corrected-to-normal vision. Participants gave informed consent before participation and received monetary compensation for their time. Experimental procedures were approved by the Central University Research Ethics Committee of Oxford. Data from all participants were retained for analysis.

#### Tasks and procedures

Tasks were programmed in Psychtoolbox (Brainard, 1997) in MATLAB. Stimuli were projected at a 60 Hz refresh rate onto a 58 × 46 cm screen that was placed ∼120 cm in front of the participant, who performed the tasks seated in the chair of the MEG.

**Figure 1.**
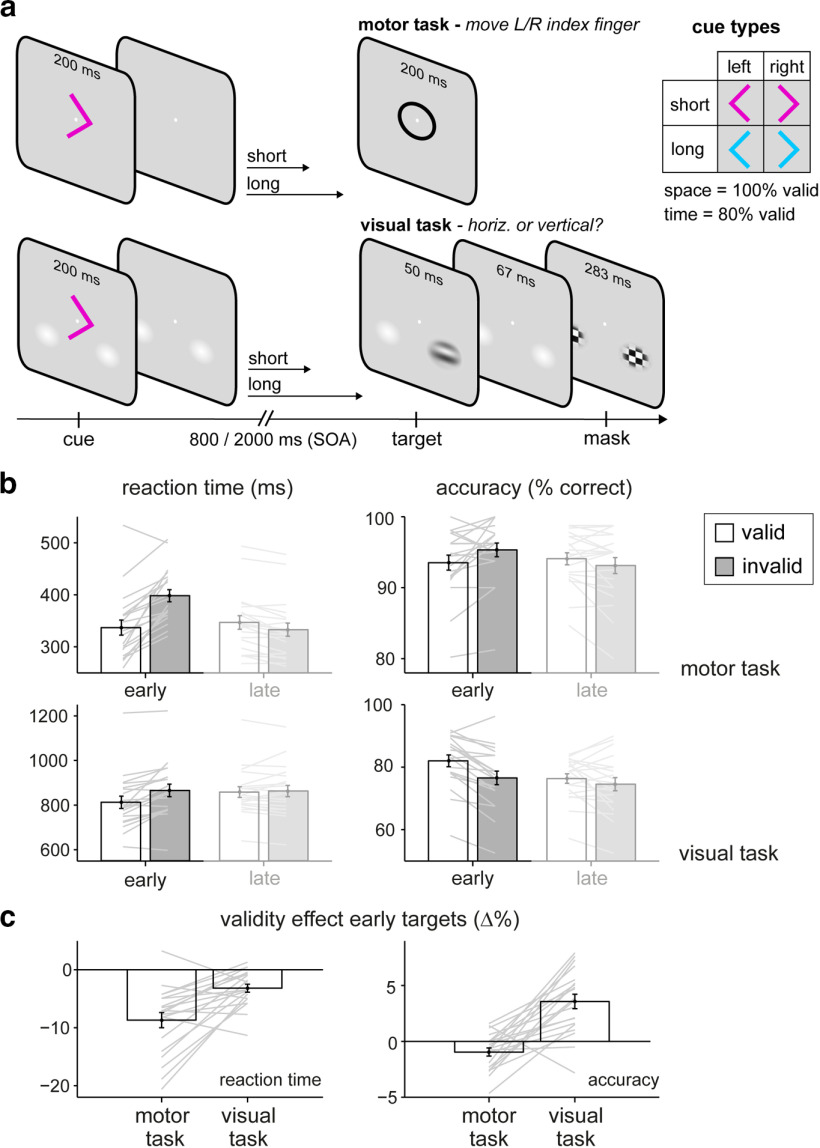
Spatial-temporal expectations benefit vision and action in distinct ways. ***a***, In the motor task, participants performed a speeded response to the central “o” target (or refrained from responding when the target was an “x” instead; 20% no-go trials). In the visual task, participants judged whether a lateralized visual target (masked by bilateral checkerboards) was horizontal or vertical. The same spatial-temporal cues (top right) preceded targets in both tasks. Cue direction instructed the relevant response hand (motor task) or predicted the side of the visual target (visual task) with 100% validity. Cue color predicted target time with 80% validity. ***b***, Reaction times and the percentage of correct responses in the motor (top) and visual (bottom) tasks as a function of temporal cue validity. Performance to late targets is shown merely for completeness (see Materials and Methods). ***c***, Temporal cue validity effects in both tasks, expressed as a percentage change between expected versus unexpected early targets. Error bars indicate ± 1 SEM. Gray lines show individual participants.

Participants performed two versions of a cued spatial-temporal orienting task (Fig. 1*a*), referred to as the motor task and the visual task. Identical visual cues and cue validities (Fig. 1*a*, top right) were used in both tasks. Cues were presented centrally for 200 ms and were at a 2.18° visual angle in width and height, with a linewidth of 0.1°. Cue direction (left or right) indicated with 100% validity what response hand was required (motor task) or on which side the visual target would occur (visual task). Cue color (blue or pink) predicted with 80% validity the time at which the target would be presented: after a stimulus-onset-asynchrony (SOA) of 800 or 2000 ms. The same intervals were used in both tasks. The mapping of color to SOA was counterbalanced across participants but was kept consistent across both versions of the task. Participants were explicitly informed about color-to-interval mappings and familiarized themselves with these mappings in practice trials in which the cues were 100% valid. Trials began with the onset of a fixation dot (0.88°) that appeared after an intertrial interval of 1500 ± 250 ms and that preceded cue onset by 750 ± 250 ms.

In both tasks, participants held their index fingers on two fiber-optic response boxes (custom made by the O.V. Lounasmaa Laboratory, Aalto University, Espoo, Finland) and responded by lifting the index finger of the left or right hand.

In the motor task, participants were instructed to lift the index finger of the cued hand as quickly as possible after a central “o” stimulus (6.4° diameter) appeared around fixation. We inserted 20% of trials with an “x” stimulus instead and instructed participants to refrain from responding in these trials (which they did successfully in a mean ± SEM of 74.63 ± 4.17% of trials). No-go trials were included to ensure that participants waited for the target to appear before making a response. In the motor task, target stimuli stayed on the screen for 200 ms.

In the visual task, the target consisted of a horizontally or vertically tilted Gabor patch (diameter, 1.96°; spatial frequency, 2 cycles/°) presented at the cued location to the left or right of fixation on top of one of two faint luminance pedestals (10% contrast). Pedestals and targets were anchored at 4.78° below the horizontal meridian at a distance of 3.38° from the vertical meridian. Target stimuli were presented for 50 ms and masked by bilateral checkerboard stimuli after an SOA of 117 ms. Masks were presented for 283 ms and were created by applying a Gaussian vignette to the convolution of 100% square-wave gratings at the two possible target orientations. Participants were instructed to report as accurately and as quickly as possible whether the grating was horizontal or vertical using the left and right index fingers. Responses in this task, however, were not linked to the direction of the cue, but to the identity of the visual target (horizontal or vertical, which was varied independent of cue direction and, consequently, of target location). The mapping between target identity and response hand was counterbalanced across participants.

One week before the first MEG session, participants came in for a 90 min session that allowed us to staircase the difficulty of the visual task. First, they familiarized themselves with the task across five blocks of 120 trials, in which we gradually decreased the time of target presentation down to 50 ms. We then applied an adaptive staircase procedure (as described in the study by Rohenkohl et al., 2014) in which we titrated the contrast of the Gabor patch contrast until discrimination was performed at 75% accuracy. During the staircase session, temporal cues were 100% predictive of the target time.

We counterbalanced the order of the visual task and motor task MEG sessions across participants. Each session contained 800 trials, distributed across four blocks of ∼15 min each. Participants could take self-paced breaks after every 40 trials. Participants came into the laboratory at separate days to complete each session, with sessions typically being <1 week apart, but never >20 d apart.

#### Analysis of behavioral data

We considered the following two dependent variables in both tasks: reaction time (RT) and percentage of correct responses. Reaction times were considered only for trials in which a response was recorded in the 200–2000 ms response window (defined relative to target onset). For analysis of the motor task data, we included both go and no-go trials for calculating accuracy (but only go trials for reaction time). We obtained the same pattern of behavioral accuracy results and statistical inferences when only go-trials were included, unless explicitly specified otherwise in our Results section.

We decided a priori to focus our analysis on the most informative data. To this end, we exclusively compared performance to early targets when these were expected (valid) versus unexpected (invalid). In cued temporal orienting tasks, it is well established (Nobre, 2001; Nobre and van Ede, 2018) that temporal cueing benefits are much more profound for early than late targets (once the early interval passes, participants can update their expectations, washing out cue validity effects for late targets). Focusing on the early targets not only simplified our analyses and increased sensitivity, it also allowed for a better comparison to the MEG analyses which, for the same reason, focused on the early interval after the cue. For completeness and transparency, we show performance to late targets in our Results section.

Because our tasks were designed to vary in overall task demands—emphasizing either response speed or perceptual accuracy—we expected large differences in overall performance between tasks, which were not our main focus. Instead, we decided a priori to focus our main statistical analyses on the performance benefits conferred by valid temporal expectations (at the early interval). To quantify the relative benefits of temporal predictions in a manner that accounted for the large global differences in RT and accuracy between tasks, we expressed validity effects as the normalized percentage change of each dependent variable [i.e., (valid – invalid)/(valid + invalid)) * 100]. We thus used paired-samples *t* tests as our primary statistical evaluation for comparing cue validity effects between tasks. For completeness, we also report the results from a repeated-measures ANOVA with the factors “task” and “validity,” which also enabled us to formally evaluate the anticipated main effects of task.

As measures of effect size, we used Cohen's *d* for all reported *t* tests, and partial η^2^ values for the ANOVA.

#### MEG acquisition and analysis

We acquired magnetoencephalography using a 306-channel VectorView MEG system (Elekta) that was housed in a magnetically shielded room at the Oxford Centre for Human Brain Activity. Data were acquired at a sampling rate of 1000 Hz. Head position was continuously tracked using four head position indicator (HPI) coils, placed behind the ears and on the forehead. A horizontal and a vertical electro-oculogram (EOG) were concurrently acquired by placing four Ag/AgCl electrodes surrounding the eyes.

##### MEG preprocessing.

Data were analyzed in FieldTrip (Oostenveld et al., 2011). Before loading the data into FieldTrip, data were cleaned using the Neuromag MaxFilter software version 2.0. At this stage, data from all participants were aligned to a common spatial positioning using the continuous HPI data, and an independent component analysis (Bell and Sejnowski, 1995) was applied to remove components associated with eye movement and blinks as detected by the EOGs. All subsequent analyses in FieldTrip were performed on the 204 planar-gradiometer channels that we combined into 102 combined-planar channels using the singular value decomposition method. Trials with excessive variance were identified and removed following visual inspection using the function “ft_rejectvisual” with the summary method. After trial removal, mean (±SEM) of 763 ± 11 (motor task) and 716 ± 28 (visual task) trials were retained for analysis.

##### Predefined channel selection.

To increase the sensitivity of our analyses, we focused our analyses on data in predefined clusters of left and right posterior (visual) and central (motor) MEG channels. Channels were chosen based on prior MEG studies from our laboratory that have consistently implicated the same set of posterior and central channels for capturing neural activity related to lateralized manual actions and lateralized visual stimuli (Heideman et al., 2018a,b, 2020). Specifically, we included the following four (combined) channels in each cluster: left central (motor): {'MEG0412 + 0413','MEG0422 + 0423','MEG0432 +0433','MEG0442 + 0443'}; right central (motor): {'MEG1112 + 1113','MEG1122 + 1123','MEG1132 + 1133','MEG1142 + 1143'}; left posterior (visual): {'MEG1912 + 1913','MEG1922 + 1923','MEG1942 +1943','MEG2042 + 2043'}; and right posterior (visual): {'MEG2032 + 2033','MEG2312 + 2313','MEG2322 + 2323','MEG2342 + 2343'}.

**Figure 2.**
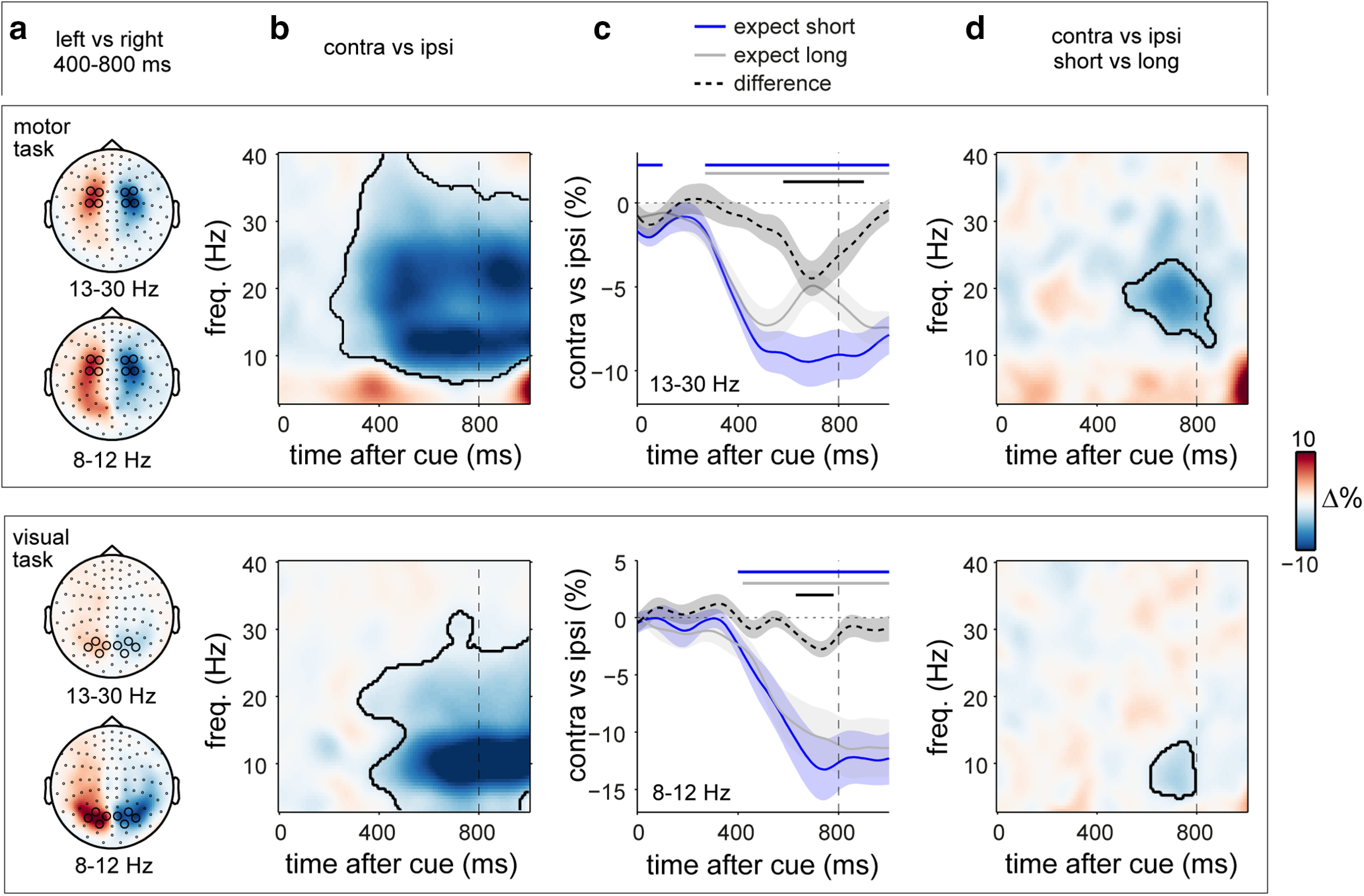
Temporal expectations latch onto modality- and frequency-specific substrates of spatial anticipation. ***a***, Topographies of power differences following left- versus right-directing cues in the 8–12 Hz alpha bands and 13–30 Hz beta bands, separately for the motor (top) and visual (bottom) tasks (collapsed over short and long cues). ***b***, Time–frequency plots of the neural lateralization in ***a***, showing the difference in spectral power contralateral versus ipsilateral to the cued side, extracted over the predefined left and right channel clusters depicted in ***a***. Black outlines indicate significant clusters following permutation analyses (Maris and Oostenveld, 2007). ***c***, Time courses of neural lateralization in ***b***, separated by temporal expectation conditions. Shadings indicate ± 1 SEM. Horizontal lines indicate significant clusters. ***d***, Time–frequency representation of the difference in neural lateralization when the target was expected early versus late. Vertical dashed lines indicate the time at which early targets would occur.

While we selected these channel clusters based on independent data, we could confirm their validity and appropriateness in the current data (Fig. 2*a*, overlay of these channel clusters on the relevant left vs right topographies). For the analysis of nonlateralized visual and motor activity, we included one additional posterior channel that was not part of the left/right channel clusters because it was a midline channel: {'MEG2112 + 2113'}.

##### Spectral analyses.

We calculated spectral power using a short-time Fourier transform with a sliding time window of 300 ms on Hanning-tapered data. The analysis window was advanced over the data in steps of 10 ms. Power was calculated for frequencies between 3 and 40 Hz in steps of 0.5 Hz. Power values were contrasted between conditions and were expressed as a percentage change: ((condition A – condition B)/(condition A + condition B)) * 100. To depict the topographies associated with relevant condition comparisons (i.e., left vs right; short vs long), we focused on the predefined 8–12 Hz (alpha) and 13–30 Hz (beta) frequency bands in the predefined 400–800 ms anticipatory (i.e., pretarget) window. For the comparison between contralateral and ipsilateral power, we compared activity following left- and right-directing cues, separately for left and right channel clusters, and subsequently pooled the contralateral versus ipsilateral contrasts between them. For our main analysis, we used the predefined central (motor) channels to calculate contralateral versus ipsilateral contrasts in the motor task, and the predefined posterior (visual) channels to calculate contralateral versus ipsilateral contrasts in the visual task.

##### Statistical analyses of MEG data.

Statistical analyses focused on contrasted time–frequency maps extracted over the predefined central (motor) and posterior (visual) channel clusters; as well as on extracted time courses collapsed over the predefined 8–12 Hz alpha band (visual task lateralization data) and 13–30 Hz beta band (motor task lateralization data). To bypass the multiple-comparisons problem, we used a nonparametric cluster-based permutation approach (Maris and Oostenveld, 2007), and used the Fieldtrip default cluster settings with 10,000 permutations. The majority of our cluster-based permutation tests clustered over the time dimension using predefined frequency bands and channels. In some cases, however, cluster-based permutation tests clustered over both time and frequency. We compared time courses of spatial lateralization following short and long cues, as well as for conditions separated by performance to early targets. Performance sorting for reaction time was performed using a median split within each participant. We focused all MEG analyses on the early interval after the cue where temporal expectation effects are known to be most pronounced (see Analysis of behavioral data, above). For the motor task brain behavior analysis sorted by accuracy, 2 (of 20) participants had an insufficient number of incorrect trials (<5 incorrect trials) to be included (compared with 25.06 ± 4 incorrect trials/participant in the remaining sample of 18 participants).

## Results

Twenty healthy human volunteers participated in two spatial-temporal anticipation tasks (Fig. 1*a*) while we recorded their brain activity using MEG. The two tasks were performed during separate visits in counterbalanced order. In the motor task, participants responded as quickly as possible to the appearance of a central “o” stimulus. In the visual task, participants discriminated whether the tilt of a masked visual stimulus was horizontal or vertical. Cues were identical in the two tasks and enabled anticipation in space and time (Fig. 1*a*, right). Cue direction informed with 100% validity that the response hand (motor task) or visual stimulus (visual task) would be left or right. Cue color informed whether the target would most likely (80% valid) appear after a short or a long cue–target interval (800 or 2000 ms after cue onset).

### Spatial-temporal expectations benefit vision and action in distinct ways

We first ascertained that participants used the temporal cue information to their benefit in both tasks. Figure 1*b* shows performance in the motor (top) and visual (bottom) tasks, as a function of whether targets occurred at the cued time (valid) or not (invalid). Focusing on early targets—where temporal cueing effects are known to be captured most sensitively (Nobre, 2001; Nobre and van Ede, 2018)—we found that valid temporal expectations yielded significantly faster responses (Fig. 1*b*, left) in both the motor task (*t*_(19)_ = −6.457, *p* = 3.448e-6, *d* = −1.444) and the visual task (*t*_(19)_ = −4.576, *p* = 2.061e-4, *d* = −1.023). For accuracy (Fig. 1*b*, right), valid temporal expectations also improved performance for early targets on the visual task (*t*_(19)_ = 4.990, *p* = 1.128e-5, *d* = 1.116) but made participants slightly worse in the motor task (*t*_(19)_ = −2.541, *p* = 0.019, *d* = −0.568), possibly reflecting a shift in the speed–accuracy trade-off in this task where overall accuracy was very high. This reduction in accuracy in the motor task was driven by a larger number of responses to valid versus invalid early no-go targets (*t*_(19)_ = 4.236, *p* = 4.467e-4, *d* = 0.947); when only considering early go targets, valid temporal expectations were associated with better performance (*t*_(19)_ = 2.216, *p* = 0.039, *d* = 0.496).

For completeness, we also ran an ANOVA with the factors cue validity and task (again focusing on data from early targets). This confirmed a significant main effect of cue validity on both RT (*F*_(1,19)_ = 47.79, *p* = 1.365e-6, η*_p_^2^* = 0.797) and accuracy (*F*_(1,19)_ = 9.537, *p* = 0.006, η*_p_^2^* = 0.275). However, this also showed robust main effects of task on both dependent variables, whereby responses in the motor task were generally much faster (*F*_(1,19)_ = 279.288, *p* = 8.107e-13, η*_p_^2^* = 0.996) and more accurate (*F*_(1,19)_ = 68.452, *p* = 1.013e-7, η*_p_^2^* = 0.968) than in the visual task. To deal with these vast differences in average RT and accuracy between tasks, we therefore expressed the validity effect in each task as a relative (percentage) change before comparing validity effects between tasks, to which we turn next (Fig. 1*c*).

In the two tasks, the same participants viewed the same cues followed by the same intervals and target probabilities; thus enabling equivalent degrees of temporal anticipation. Yet, we observed remarkably distinct patterns of performance benefits between tasks (Fig. 1*c*). Temporal expectations conferred significantly larger reaction time benefits to early targets in the motor task than in the visual task [Fig. 1*c*, left; 8.69% (motor) vs 3.17% (visual) faster responses; *t*_(19)_ = −4.143, *p* = 5.525e-4, *d* = −0.926], but significantly larger accuracy benefits to early targets in the visual task than in the motor task [Fig. 1*c*, right; −0.87% (motor) versus 3.34% (visual) more accurate responses; *t*_(19)_ = −5.441, *p* = 2.998e-5, *d* = −1.217; and this was also the case when only including early go trials for the motor task; *t*_(19)_ = −3.752, *p* = 0.001, *d* = −0.839].

Thus, the nature of the benefits of temporal foreknowledge on performance are purpose dependent: affecting predominantly reaction time when perceptual demands are low and speed is of the essence (motor task), and, conversely, accuracy when the perceptual demands are high and response speed is less important (visual task).

### Temporal expectations latch onto modality-specific substrates of spatial anticipation

In both tasks, temporal expectations occurred alongside of valid foreknowledge about space—whether the response was required with the left or right hand (motor task) or whether the visual target would appear left or right on the screen (visual task).

**Figure 3.**
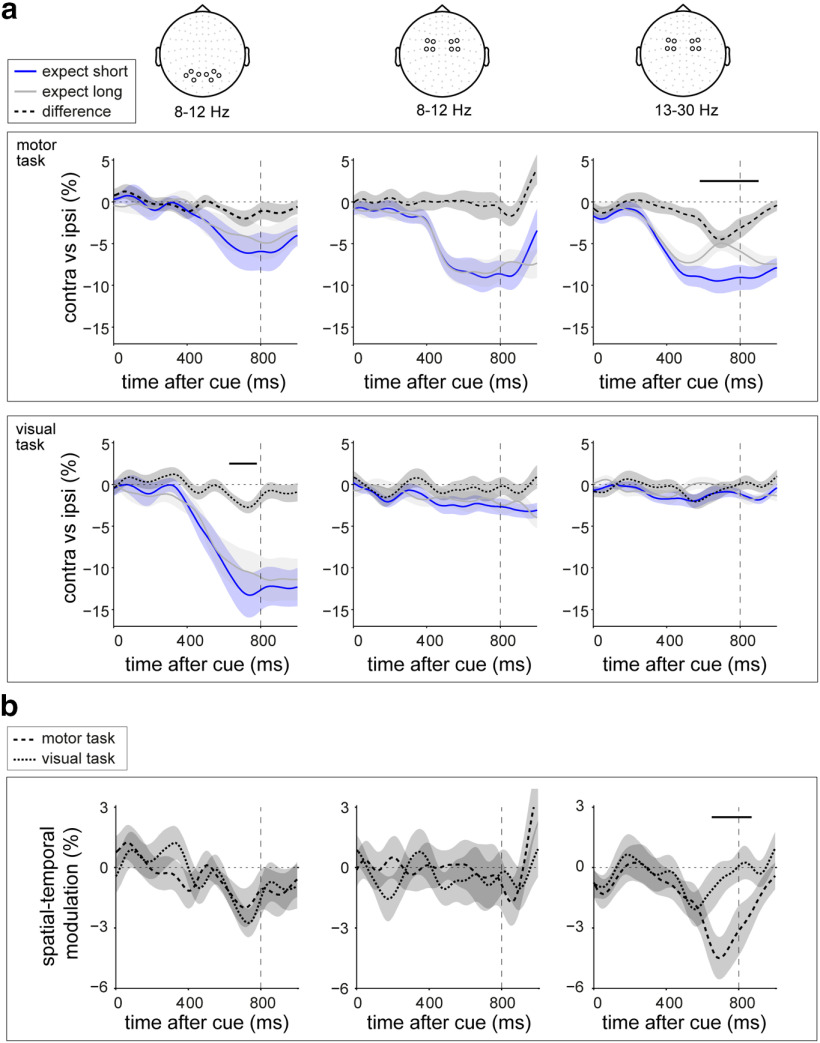
Spatial and spectral specificity of spatial-temporal neural modulations for vision and action. ***a***, Time courses of neural lateralization in posterior 8–12 Hz activity (left), central 8–12 Hz activity (center), and central 13–30 Hz activity (right) in the motor (top) and the visual (bottom) tasks. ***b***, Direct task comparison of the observed spatial-temporal modulations in ***a***. Shadings indicate ± 1 SEM. Black horizontal lines indicate significant clusters of the modulation in neural lateralization by temporal expectation (***a***) or their difference between tasks (***b***). Top right and middle left panels are identical to those in Figure 2*c*.

To characterize the neural dynamics of spatial anticipation in our tasks, we collapsed over short and long cues and contrasted neural activity following left- versus right-directing cues. This confirmed clear lateralization of neural activity in the 8–12 Hz alpha band and the 13–30 Hz beta band in both tasks (Fig. 2*a*), which is consistent with many prior studies (Pfurtscheller and Berghold, 1989; Worden et al., 2000; Schoffelen et al., 2005; Thut et al., 2006; Donner et al., 2009; Jensen and Mazaheri, 2010; Gould et al., 2011, 2012; van Ede et al., 2011; Kizuk and Mathewson, 2017; Heideman et al., 2018a,b, 2020; Boettcher et al., 2020). As expected, the topographical distribution and spectral content of these spatial anticipation signatures were distinct between tasks. In the motor task, the lateralization was most pronounced in central (putative motor) MEG channels, and the neural modulation was pronounced across the 8–30 Hz band (Fig. 2*a*,*b*, top row; cluster, *p* < 0.0001). In contrast, in the visual task the lateralization was most pronounced in posterior (putative visual) MEG channels, and was particularly pronounced in the 8–12 Hz alpha band (Fig. 2*a*,*b*, bottom row; cluster, *p* < 0.0001; Fig. 3).

Critically, these signatures of spatial anticipation were each sensitive to concurrent temporal expectations (Fig. 2*c*). In the motor task, the attenuation of beta activity in contralateral (vs ipsilateral) motor electrodes was significantly stronger in the early interval after the cue, if participants expected the target to occur early (Fig. 2*c*, top row; cluster, *p* = 0.0034). Likewise, in the visual task, the contralateral attenuation of posterior alpha activity was significantly stronger in the early interval after the cue, if participants expected the visual target to occur early (Fig. 2*c*, bottom row; cluster, *p* = 0.0123). A time- and frequency-resolved plot of the difference in spatial anticipation following short versus long cues (Fig. 2*d*) confirmed the spectral specificity of these spatial-temporal anticipation effects.

To assess the spatial and spectral specificity of these purpose-dependent spatial-temporal neural modulations, we additionally considered, separately for each task, alpha modulations in posterior channels (Fig. 3*a*, left), as well as alpha and beta modulations in central channels (Fig. 3*a*, right and middle). A direct comparison between tasks in these spatial-temporal modulations (Fig. 3*b*) revealed a significantly stronger spatial-temporal modulation of central beta activity for the motor versus the visual task (Fig. 3*b*, right; cluster, *p* = 0.006). In contrast, the spatial-temporal modulation of posterior alpha oscillations—while significant in the visual task, but not in the motor task (Fig. 3*a*, left)—did not survive the direct task comparison (Fig. 3*b*, left). This is likely because of the observation that a trend for a similar spatial-temporal modulation of posterior alpha oscillations was observed in the motor task (possibly reflecting visual imagery of using the left vs right hand). Moreover, in contrast to the highly robust spatial-temporal modulation of beta activity in the motor task, the spatial-temporal modulation of posterior alpha lateralization in the visual task was less pronounced overall.

Thus, in the presence of spatial foreknowledge, temporal expectations latch onto and modulate neural signatures of spatial anticipation, in a modality- and frequency-dependent manner. This was particularly clear for the central beta modulation, which was only observed (and significantly stronger) in the motor task.

### Anticipatory modulations covary with speed in the motor task but accuracy in the visual task

**Figure 4.**
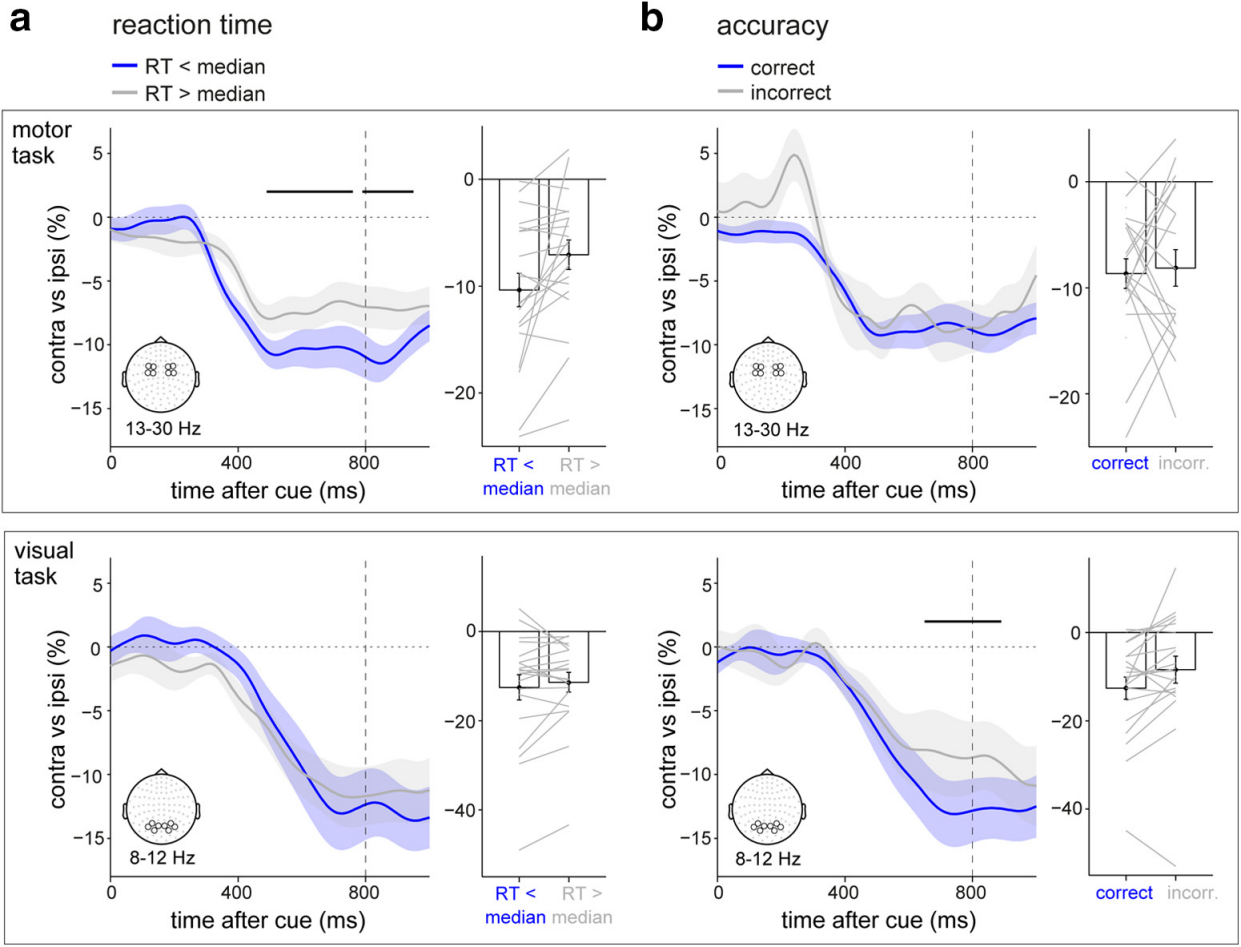
Anticipatory modulations predict speed in the motor task but accuracy in the visual task. ***a***, ***b***, Neural lateralization in central channels in the motor task and posterior channels in the visual task (compare Fig. 2*c*) separated by reaction times (***a***) or accuracy (***b***). Only trials with an early target were considered. Horizontal black lines indicate significant clusters of the difference between trials with “good” and “bad” performance. Bar graphs show the lateralization extracted over the significant windows in Figure 2*c* (black horizontal lines) and serve primarily to depict the participant distribution of the effects. Gray lines show individual participants. For the accuracy-sorted data in the motor task, 2 (of 20) participants had insufficient incorrect trials to be included. Shadings and error bars indicate ± 1 SEM.

Separating the data by performance to the early targets also revealed how the anticipatory modulation of central beta oscillations in the motor task was associated with faster responses (Fig. 4*a*, top; cluster, *p* values = 0.0042, 0.0186) but had no systematic relation to response accuracy (Fig. 4*b*, top). While we noted how incorrect trials were associated with an initial increase in contralateral versus ipsilateral beta activity (i.e., early lateralization in the “wrong” direction; Fig. 4*b*, top), this effect did not survive cluster-based permutation testing and possibly reflects noise, because of the limited number of incorrect trials in the motor task.

In contrast, the anticipatory modulation of posterior alpha oscillations in the visual task was associated with more accurate perceptual discrimination (Fig. 4*b*, bottom; cluster, *p* = 0.0137), but showed no systematic relation to response times (Fig. 4*a*, bottom). This is consistent with the observation that the cues primarily affected reaction time in the motor task, but accuracy in the visual task (Fig. 1*c*); and shows that such brain–behavior associations too, may be fundamentally purpose dependent.

### Temporal orienting for action is additionally associated with posterior alpha attenuation

**Figure 5.**
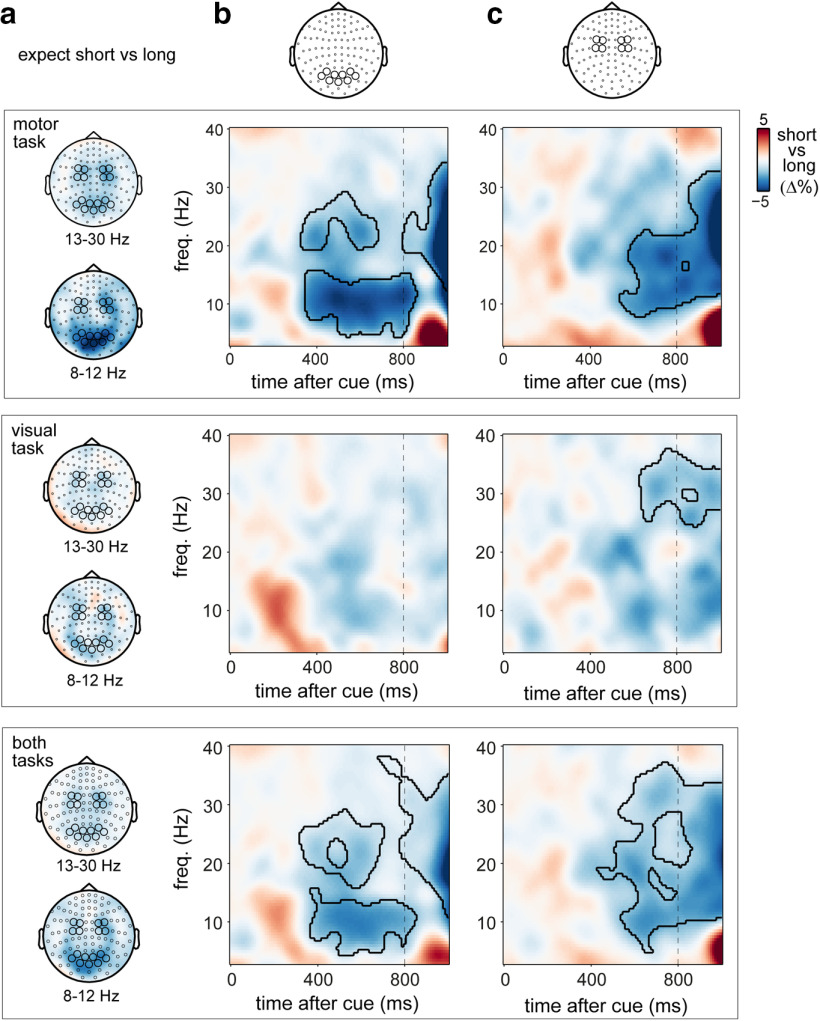
Global temporal expectation effects in posterior and central channels across both tasks. ***a***, Topographies of the power difference following short versus long cues in the 8–12 Hz alpha and 13–30 Hz beta bands, separately for the motor (top) and visual (middle) tasks (collapsed over left and right cues), as well as the main effect of temporal expectation collapsed across both tasks (bottom). ***b***, ***c***, Time–frequency plots short versus long difference in the preselected posterior-visual (***b***) and central-motor (***c***) channels in both tasks. Black outlines indicate significant clusters.

We set up our tasks such that demands were largest for action in the motor task and largest for vision in the visual task. At the same time, both were “visual–motor” tasks with visual targets and manual responses. We therefore also looked for general temporal orienting effects (collapsed over left/right spatial expectations) in posterior-visual and central-motor channels in both tasks. As shown in Figure 5, this revealed particularly clear temporal expectation modulations in the motor task (Fig. 5, top). Despite the fact that the visual “target” in the motor task was always clearly visible and easy to discriminate, we found a robust attenuation of alpha activity in the posterior MEG channels following cues predicting a short versus long interval (Fig. 5*b*, top; cluster, *p* = 0.0089). This effect in the 8–12 Hz alpha band had a clear posterior topography (Fig. 5*a*, 8–12 Hz topography in motor task) concentrating in the same channels that also showed the clearest alpha lateralization in the visual task **(**Fig. 2*a*). A similar, albeit weaker, posterior modulation appeared in the visual task (Fig. 5*b*, middle), though no clusters survived statistical evaluation. When collapsing across tasks to look at the main effect of temporal expectation, this posterior alpha modulation did survive (Fig. 5*b*, bottom; cluster, *p* = 0.015).

In both tasks, we also observed similar alpha/beta attenuation in central sites (Fig. 5*c*; motor task cluster, *p* = 0.0044; visual task cluster, *p* = 0.0158). This became particularly clear when looking at the main effect of temporal expectation, collapsed across tasks (Fig. 5*c*, bottom row; cluster, *p* = 0.008). For the motor task, it is possible that this central modulation may directly reflect the contralateral beta attenuation associated with spatial-temporal expectations (Figs. 2, 3). However, the fact that a similar modulation appears in the visual task (where we did not see any spatial-temporal modulation in central channels) opens the possibility that these global modulations of temporal expectation may occur on top of the spatially specific signatures reported in the preceding figures.

## Discussion

We investigated the behavioral consequences and anticipatory neural dynamics of cued spatial-temporal expectations in service of distinct task demands. This revealed how identical temporal cues can have drastically different consequences on performance—and be mediated by distinct neural modulations—when the task requires a speeded response versus a demanding perceptual judgment. The modulatory consequences of temporal expectation on neural activity and behavior thus not only depend on the source of temporal expectations, but also on its anticipated purpose (as also discussed in Shalev et al., 2019).

Our results provide a compelling reason to abandon attempts to pin down “the” way in which temporal expectations facilitate performance. In the past, numerous studies asked whether temporal expectations modulated early sensory stages of visual processing or were confined to modulate only late, response-related processes (Miniussi et al., 1999; Griffin et al., 2002; Correa et al., 2006; Lange et al., 2006; Lange and Röder, 2006). By equating the appearance and temporal probabilities of informative cues and manipulating the perceptual versus motor nature of perceptual demands, we show that temporal expectations operate in a contextually relevant manner by latching onto and optimizing other anticipatory biases afforded within a task, and predominantly affecting performance variables that are most relevant to the task. To demonstrate this, in our study, we kept the nature of temporal expectations fixed, using associative temporal cues, and manipulated only the task demands. Further flexibility in the modulatory consequences of temporal expectations have been proposed when different sources of expectations are compared (e.g., associative vs rhythmic; Rohenkohl and Nobre, 2011; Triviño et al., 2011; de la Rosa et al., 2012; Breska and Deouell, 2014; Morillon et al., 2016; Breska and Ivry, 2018; Nobre and van Ede, 2018; Bouwer et al., 2020).

In the current work, we focused on the modulatory consequences of temporal expectation (on both neural activity and on behavioral performance). As such, our data leave open the question of whether the origins of temporal expectations are purpose dependent as well. It remains a theoretical possibility that common sources of temporal expectation yield distinct modulatory consequences as a function of (anticipated) task demands.

By studying temporal expectations in the context of concurrent spatial expectations, our MEG data demonstrated how temporal expectations can latch onto existing neural modulations in a modality- and frequency-dependent manner (but see Fischer et al., 2013; Pomper et al., 2015), which was particularly clear for the spatial-temporal modulation of central beta activity that was observed exclusively in the motor task. These results are consistent with several prior demonstrations of such spatial-temporal modulation of oscillatory brain activity in service of perception (Rihs et al., 2009; Lima et al., 2011; van Ede et al., 2011; Heideman et al., 2020) and action (Schoffelen et al., 2005; Heideman et al., 2018b, 2020), including within the context of working memory (van Ede et al., 2017; Boettcher et al., 2020). Building on these studies, which targeted a single goal in isolation, the current study demonstrates the purpose-dependent manifestation of such spatial-temporal expectations within a single experiment, using the same cues and the same participant sample across the two tasks.

Action anticipation of the left/right response hand was associated with a spatially specific attenuation of activity that spanned the alpha and beta bands (Pfurtscheller and Berghold, 1989; McFarland et al., 2000; Donner et al., 2009; Boettcher et al., 2020). Yet, in the same motor task, the temporal modulation of this spatial anticipation signature appeared confined to the beta band. Thus, while sensorimotor alpha and beta activity may both contribute to effector-specific action preparation, beta activity appears particularly susceptible to time (consistent with more pronounced temporal modulation of anticipatory beta attenuation; van Ede et al., 2011; Todorovic et al., 2015). The absence of the spatial-temporal alpha modulation is unlikely to be accounted for by differences in the temporal sensitivity of alpha oscillations or our analyses hereof. For example, we observed timed modulation of alpha lateralization in the visual task (see also Rohenkohl and Nobre, 2011; Zanto et al., 2011; van Ede et al., 2017). Moreover, next to the central modulation in lateralized beta activity, in the same motor task we observed a pronounced and spatially global modulation of posterior alpha activity. However, unlike the spatial-temporal beta modulation in central sites, we propose that this posterior alpha modulation may not relate to motor preparation directly but may, instead, relate either to visual anticipation of the visual go signal in this task, though another possibility is that this more global signature reflects the engagement of parietal cortices associated with the control of attention.

It is tempting to attribute the spatial-temporal alpha versus beta modulations to anticipation of perception versus action, respectively. However, similar beta attenuation was previously documented during spatial-temporal somatosensory anticipation (van Ede et al., 2011). It may thus be more appropriate to attribute the frequency-specific nature of the spatial-temporal anticipation signatures to the respective brain areas/networks involved (visual/parietal vs sensorimotor), rather than to a hard distinction between perception and action per se.

Our data also show that the relation between anticipatory neural modulations and performance (for review, see Ergenoglu et al., 2004; Thut et al., 2006; Hanslmayr et al., 2007; van Dijk et al., 2008; Mazaheri et al., 2009; Gould et al., 2011; Haegens et al., 2011; van Ede et al., 2012a,b, 2017) are contingent on task demands too. In our data, the degree of sensorimotor beta attenuation in the motor task predicted reaction times (but not accuracy; though we note that there were very few incorrect trials available in this task), while the degree of posterior alpha attenuation in the visual task predicted accuracy (but not reaction times). In the domain of perception, most previous reports have linked posterior alpha oscillations to measures of detection performance (hit rate) and reaction times. On this basis, it has been suggested that such “states” of attenuated alpha activity may primarily be associated with enhanced cortical excitability and thereby make you more likely to see a stimulus (criterion shift), without affecting the quality or accuracy of perception (Iemi et al., 2017; Benwell et al., 2018). We used a demanding backward-masked two-alternative discrimination task and show that, in such a demanding perceptual discrimination task, alpha can be related (be it in a correlational sense) to perceptual accuracy. These brain–behavior associations are likely to reflect (at least in part) variability in the use of temporal expectations.

Complementing the temporal expectation-dependent amplification of spatial anticipation; we also found evidence for more global effects of temporal expectation. Most strikingly, we observed a robust posterior alpha attenuation in the motor task. While this likely reflects visual anticipation of the target in this task, this modulation is nevertheless remarkable as the visual target was very easy to see and discriminate. It does, however, beg the following question: why was this posterior modulation even more apparent in the motor task than in the visual task, in which visual demands were much higher? Possibly, this global modulation may be stronger when anticipating a central foveal visual target, which was the case only in our motor task.

Another possibility, however, is that there is greater motivation for relying on temporal expectations in the speeded response task, which injected urgency for completing sensorimotor processing as rapidly as possible. This would, in turn, lead to more profound neural modulations. In line with this, like the global posterior modulation, the identified spatial-temporal modulation also appeared more profound in the motor task (in central sites) than the corresponding spatial-temporal modulation in the visual task (in posterior sites). Such a purpose-dependent incentive to use temporal foreknowledge remains an interesting possibility for further investigation.

In our experiment, response speed was most important in the motor task, while accuracy was most important in the visual task. We do not intend to claim that motor anticipation will only ever influence response speed while visual anticipation will only ever influence accuracy—and likewise that their neural correlates are only relevant for either measure of performance. Instead, we emphasize that modulatory consequences may inevitably depend on task demands. Our tasks were set up to emphasize speed or accuracy in motor and visual tasks, respectively. In future work, complementary tasks could be devised that would emphasize accuracy in the motor task, but speed in the visual task. In such cases, the consequences of temporal expectations—and their neural modulations—on speed and accuracy may well reverse in their relation to perception and action.
